# Impacts of fire severity and exotic invasion of *Pinus radiata* on post-fire regeneration of relict *Nothofagus alessandrii* forests in central Chile

**DOI:** 10.3389/fpls.2026.1772087

**Published:** 2026-04-16

**Authors:** Claudia Leal-Medina, Mauricio Galleguillos, Javier Lopatin, Rocío Urrutia-Jalabert, Mauro E. González

**Affiliations:** 1 Escuela de Graduados, Facultad de Ciencias Forestales y Recursos Naturales, Universidad Austral de Chile, Valdivia, Chile; 2 Sensor-based Geoinformatics, Faculty of Environment and Natural Resources, University of Freiburg, Freiburg im Breisgau, Germany; 3 Data Observatory Foundation, Santiago, Chile; 4 Center for Climate and Resilience Research (CR)2, University of Chile, Santiago, Chile; 5 Departamento de Ingeniería Hidráulica y Ambiental, Pontificia Universidad Católica de Chile, Santiago, Chile; 6 Departamento de Ecosistemas y Medio Ambiente, Pontificia Universidad Católica de Chile, Santiago, Chile; 7 Faculty of Engineering and Science, University Adolfo Ibáñez, Santiago, Chile; 8 Departamento de Ciencias Forestales, Facultad de Ciencias Agropecuarias y Medioambiente, Universidad de la Frontera, Temuco, Chile; 9 Millenium Nucleus of Patagonian Limit of Life (LiLi), Valdivia, Chile; 10 Laboratorio de Dendrocronología y Cambio Global, Instituto de Conservación, Biodiversidad y Territorio, Facultad de Ciencias Forestales y Recursos Naturales, Universidad Austral de Chile, Valdivia, Chile; 11 Laboratorio de Ecología de Bosques, Instituto de Conservación, Biodiversidad y Territorio, Facultad de Ciencias Forestales y Recursos Naturales, Universidad Austral de Chile, Valdivia, Chile; 12 Center for Fire and Socioecosystem Resilience (FireSES), Valdivia, Chile

**Keywords:** ecosystem vulnerability, endangered species, invasive species, *Pinus radiata*, post-fire regeneration

## Abstract

In Chile, wildfires caused mainly by human activity have led to substantial changes in forest composition and structure. These disturbances may promote irreversible forest degradation, particularly when critically endangered forests are affected by high intensity fires and invasion of exotic species. Understanding post-fire regeneration dynamics in endangered *Nothofagus alessandrii* forests, and the invasion of *Pinus radiata* under varying fire severities, is crucial to ensure the persistence of these ecosystems. This study examined the early post-fire response of *N. alessandrii* forest fragments embedded within a *P. radiata* plantation matrix following the 2017 ‘Las Máquinas’ megafire in central Chile. Fire severity was assessed using the differenced Normalized Burn Ratio (dNBR) derived from Sentinel-2 imagery. Post-fire vegetation dynamics were analyzed using time series of two spectral indices (NDVI and MSAVI2) from 2018 to 2021, applying linear mixed-effects models based on PlanetScope imagery. Early post-fire responses of *N. alessandrii* forests and *P. radiata* invasion were evaluated through establishment density and tree-ring radial growth across different fire severity classes. Results showed rapid vegetation recovery in areas affected by moderate and high fire severity. Post-fire regeneration of *N. alessandrii* occurred mainly through vegetative resprouting, with higher resprouting rates observed in moderately and severely burned sites (70%) compared to low-severity areas (48%). Radial growth of *N. alessandrii* did not differ significantly between moderate and high severity sites (*p >* 0.05), while *P. radiata* showed increased growth under high fire severity and greater growth than native species in areas severely burned (*p <*0.05). A direct relationship was observed between fire severity and the degree of invasion by *P. radiata*, with high-severity sites showing the highest levels of invasion (9,760 ind/ha). These results highlight the increased vulnerability of this already endangered ecosystem to severe fires and the invasion of *P. radiata*. Both processes induce irreversible forest degradation by reinforcing a positive fire feedback loop and intensifying competition with native species in severely burned areas. These results indicate the urgent need to effectively control the *P. radiata* invasion in the burned forests of *N. alessandrii* to avoid the loss of the last remaining fragments of this threatened species.

## 1 Introduction

In recent decades, global forest cover has experienced a significant decline due to deforestation and forest degradation (FAO, 2022). In South America, the main cause of deforestation is the conversion of forested areas to agricultural and urban use (Van der Borght and Pallares Barbera, 2023; Zalles et al., 2021). In south-central Chile and northern Patagonia, forest loss and degradation have been exacerbated by large-scale wildfires triggered by extreme weather conditions, such as high temperatures and prolonged droughts (Carrasco-Escaff et al., 2024; Cordero et al., 2024; Moreno et al., 2025). The impacts of these events on the recovery capacity of relict and highly threatened ecosystems and on their adaptive potential to face climate change remain uncertain (Nolan et al., 2021).

South-central Chile is considered a global biodiversity hotspot, mainly due to its high level of endemic species (Arroyo et al., 2008). However, these species habitats are increasingly threatened, primarily due to land use changes and landscape fragmentation (Alaniz et al., 2016; Miranda et al., 2017; Lara et al., 2021). The Maulino Coastal Forest (MCF) is one of the most threatened ecosystems in the area (Arnold et al., 2009; Bustamante et al., 2005). Situated between Mediterranean and Temperate ecosystems, these transitional forests are dominated by deciduous species such as *Nothofagus glauca* (hualo) and *Nothofagus alessandrii* (ruil) listed as Vulnerable (VU) and Endangered (EN), respectively (Barstow et al., 2017; MMA, 2019).

The *Nothofagus alessandrii* forests have experienced a long-term decline in their distribution area. One of the most pronounced periods of decline occurred between 1981 and 1991, with a deforestation rate of 8.15%. This reduction is reflected in the dramatic loss of forest cover, which decreased from 824.8 to 352.2 hectares (Bustamante and Castor, 1998). Consequently, over 50% of these forests were lost, primarily replaced by *Pinus radiata* plantations, leaving isolated fragments scattered across the landscape (Bustamante and Castor, 1998; Grez et al., 1998). Recently, a significant proportion of *N. alessandrii* forests were affected by the ‘Las Máquinas’ megafire in 2017. The fire burned approximately 160,000 hectares in total and impacted nine of the fifteen remaining populations of this species (Valencia et al., 2018). Of this area, 172 hectares corresponded to *N. alessandrii* forests, representing about 55% of the total forest area of the species (Santelices-Moya et al., 2023). This extensive damage has substantially increased the vulnerability of *N. alessandrii* forests and exacerbated their threat status (González et al., 2022a).

*Pinus radiata* invasion into the MCF has been well documented, particularly in fragmented areas surrounded by pine plantations (Bustamante et al., 2003; Bustamante and Simonetti, 2005). However, after ‘Las Máquinas’ megafire in 2017, the massive invasion of seedlings of this species raised alarms about the enormous threat posed to these native forest ecosystems (González et al., 2022a; San Martín, 2022; Leal-Medina et al., 2024). *Pinus radiata* is renowned for its fire adaptations such as serotiny and high post-fire recruitment, especially in areas severely impacted by fires (Richardson and Bond, 1991; Richardson et al., 2008). Although this invasive process has been reported in different studies, the patterns, mechanisms and ecological impacts of such invasions following large-scale wildfires are still relatively unknown (Pauchard et al., 2011).

Given the highly fragmented native forest landscape dominated by *P. radiata*plantations and the recurrence of intense wildfires, the dynamics of *N. alessandrii* recovery are particularly complex (Carvajal et al., 2019; Leal-Medina et al., 2024). Understanding these post-fire processes is crucial to assess the resilience of relict forests and inform effective management strategies (González et al., 2022b, a; San Martín, 2022). Monitoring vegetation recovery and invasion dynamics requires tools that capture spatial and temporal variability across large areas (Viana-Soto et al., 2020). Remote sensing provides such capabilities, offering multitemporal observations before, during, and after disturbance events (Alegria, 2022; Gao et al., 2020). Medium and high resolution satellite imagery, such as Sentinel-2 (10–20 m) and PlanetScope (3–5 m), have proven particularly valuable for tracking vegetation dynamics after fire (Cheng et al., 2020; Santelices-Moya et al., 2022) and biological invasions (Kattenborn et al., 2019; Lopatin et al., 2019). Spectral indices derived from the reflectance in the red and near infrared bands are reliable indicators of vegetation greenness and can effectively represent forest regrowth (Wang et al., 2022).

The Normalized Difference Vegetation Index (NDVI) and the Modified Soil-Adjusted Vegetation Index (MSAVI2) are two of the most frequently used indices for quantifying the vigor and productivity of vegetation (Yan et al., 2025; Alegria, 2022). The use of medium or high resolution imagery further refines the assessment of fire disturbance impacts and forest recovery dynamics, revealing that regeneration patterns differ markedly with fire intensity and vegetation type (Wang et al., 2022). Integrating these spectral indices with field observations is essential for detecting changes in forest composition and structure, as well as for distinguishing the mechanisms that drive post-fire regeneration such as resprouting and seedling recruitment (Bond and Midgley, 2001; Pausas and Keeley, 2014). Moreover, incorporating species-specific growth responses tree-ring radial increments and height growth, yields valuable insight into post-disturbance productivity and resilience of forest recovery. The production of stem wood is strongly mediated by environmental drivers such as incident solar radiation, soil water and nutrient availability, and temperature (Álvarez et al., 2013). Collectively, these remote-sensing and dendrochronological approaches constitute a robust framework for evaluating how post-disturbance processes may shift under climate change and the increasing frequency of megafires (Nolan et al., 2021).

Frequent wildfires have the potential to disrupt the expected successional trajectories of ecosystems. This can lead to irreversible forest degradation, which is characterized by canopy loss, the dominance of invasive species, the replacement of species, and changes in forest structure (Ferreira et al., 2012; Thompson et al., 2011; Vásquez-Grandón et al., 2018). The main goal of this research was to evaluate the effects of fire and *P. radiata* invasion on the early response of *N. alessandrii* forests after the 2017 ‘Las Máquinas’ megafire. Specifically, we aimed: (1) to assess fire severity and vegetation recovery using remote sensing data and (2) to evaluate the post-fire regeneration of *N. alessandrii* along with the *P. radiata* invasion.

In this context, three primary hypotheses are proposed: (1) Vegetation vigor indices will reflect differences in post-fire vegetation recovery across fire severity classes. (2) Greater fire severity will facilitate invasion by *Pinus radiata*, resulting in higher density and growth compared with *Nothofagus alessandrii*. (3) Post-fire recruitment of *N. alessandrii* will be primarily of vegetative origin with a higher density of individuals in sites burned with greater severity.

## 2 Materials and methods

### 2.1 Study area

**Figure 1:**
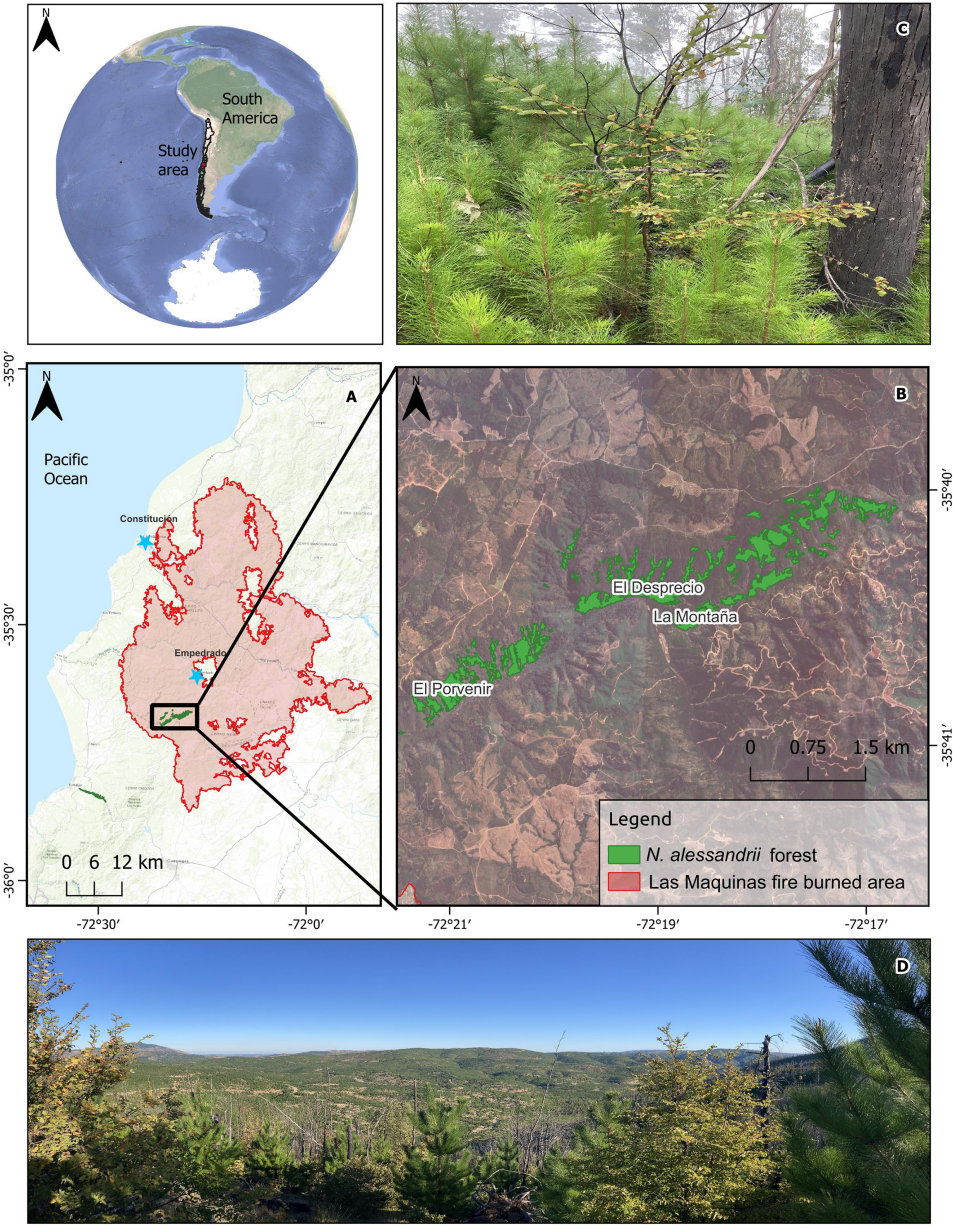
Map of the study sites: **(A)** Maule region in south-central Chile; **(B)** Remnant populations of *Nothofagus alessandrii* affected by the ‘Las Máquinas’ megafire; **(C)** Dense invasion of *P. radiata* over the *N. alessandrii* forest fragments affected by high severity; **(D)** Overview of landscape burned in 2017 after 6 years. The front view the establishment of *P. radiata* and *N. alessandrii*.

The study area was located at 35°36’ S and 72°16’W in the Coastal Range of the Maule region (**Figure 1**). Three *N. alessandrii* populations affected by ‘Las Máquinas’ megafire in 2017 were selected: ‘El Desprecio’, ‘El Porvenir’, and ‘La Montaña’ (**Table 1**). This wildfire affected the area with different fire severities, ranging from high-severity to medium and low severity combustion (Valencia et al., 2018).

**Table 1:** Location and general characteristics of the study sites in *Nothofagus alessandrii* fragments.

Geographical information		Site characteristics		
Study site	Coordinates (S, W)	Altitude (m)	Area (ha)	Landowner
El Porvenir	35°40, 72°19’	300	62.8	Forest company
La Montaña	35°40, 72°18’	350	43.2	Small landowner
El Desprecio	35°40, 72°20’	344	39.2	Forest company

The area has a Mediterranean climate with coastal influence (Csb), which is characterized by precipitation concentrated during winter and a long dry season marked by high temperatures from October to March (Beck et al., 2018). The geomorphology of the region is characterized by undulating topography, with low hills and flat tops, whose maximum height does not exceed 700 m a.s.l. Soil types are classified as Inceptisols and Alfisols (Bonilla and Johnson, 2012; Soto et al., 2019). Soil properties exhibit substantial spatial variability. Soils under native forests tend to be thicker, have higher levels of soil organic matter, and have richer invertebrate diversity than those found in tree plantations (Cifuentes-Croquevielle et al., 2020; Tolorza et al., 2024).

*Nothofagus alessandrii* forest grows in favorable microhabitats particularly in south-oriented facing slopes, which receive less solar radiation and are exposed to moist winds (San Martín, 2022). Forest fragments of this species are sparsely located in a latitudinal extension of around 100 km along the Coastal Range within a forest exotic plantation’s matrix (Torres-Díaz et al., 2007). The structure of these forests corresponds to second-growth forests, with multi-stemmed trees resulting from historical disturbances, including fires, intensive timber and fuel extraction (González et al., 2022b, a). These human activities have significantly reduced the species distribution and natural regeneration (San Martín, 2022).

### 2.2 Fire severity indices

Fire severity was assessed using the difference Normalized Burn Ratio (dNBR), a widely used index for characterizing the effects of fires (Key and Benson, 2006; Miller and Thode, 2007; Schimmel and Granstrom, 1996). The selection of dNBR was made based on the findings of Santelices-Moya et al. (2022), who evaluated dNBR, RdNBR, and NBR in all populations of *Nothofagus alessandrii* affected by the ‘Las Máquinas’ megafire in 2017. The study’s findings indicated that the three severity indices provide a comparable classification system in terms of severity assessment and post-fire response. However, El Desprecio, El Porvenir, and La Montaña sites, the dNBR index appeared to be more conservative in identifying high-severity areas, thereby reducing the risk of overestimation in cases of high damage.

The dNBR was calculated using Sentinel-2 satellite imagery level 2A atmospherically corrected and orthorectified with a resolution of 10 m by comparing the NBR before and after the fire. Images from the 10th of January 2017 (pre-fire) and from the 15th of March 2017 (post-fire) were selected. The resulting values were classified according to the general severity categories proposed by Key and Benson (2006) (**Supplementary Table 1**). We defined sampling “sites” according to severity classes to install sampling plots for each class (**Supplementary Figures 1B–D**).

Fire severity was subsequently validated through field observations using a modified approach based on the Composite Burn Index (CBI) described by Key and Benson (2006). This method enables fire severity to be assessed based on observable vegetation traits such as tree mortality, trunk blackening and vigorous vegetation cover. The CBI index was primarily used as a complementary tool, especially to define transition areas when the effects of fire are not evident. The assessment focused exclusively on canopy-related attributes because they exhibit persistent signs of fire effects or delays that could become visible within the first five years (Robbins et al., 2022). Based on these criteria, the three sites were classified into three severity categories five years after fire: a) High severity: characterized by the absence of live canopy cover, fully charred trees, and high mortality; b) Moderate severity: characterized by a partially affected total canopy cover (20–50%), structural heterogeneity, and variable vegetation damage; c) Low severity: where more than 50% of the total canopy cover remains intact and the impact of fire is mainly limited to the understory (**Supplementary Figure 2**).

To reduce the potential for underestimating burned areas, especially in low- and moderate-severity classes, the dNDBR values were complemented with field data (Santelices-Moya et al., 2022). We applied new dNBR index thresholds based on field observations derived from the Composite Burn Index (CBI) to ensure an accurate representation of fire severity (**Supplementary Figure 2**) and continuous dNBR values. This reclassification aimed to improve the accuracy of fire severity detection per ploy and simplifying the original five severity classes into three broader classes. Additionally, this approach simplify the identification of transitional areas between severity classes.

A descriptive statistical analysis was then performed using the dNBR values to define new thresholds for fire severity reclassification. Statistical measures, including the mean, median, and quartiles, were calculated, and a frequency histogram was used to explore the distribution of values. The first and third quartiles were selected as cut-off points to define low, moderate, and high fire severity classes, enabling a more accurate reclassification of fire severity across the study area (**Supplementary Table 2**; **Supplementary Figure 3**).

### 2.3 Field data

The assessment of the structure and composition of the burned *N. alessandrii* forests was conducted between June and September 2022, with a total of 18 plots. The sampling design initially intended was a random sample design according to fire severity classes. However, due to access limitations in the study area, which include a south-west facing slope with an average slope of 20-30° in the steepest zones (**Supplementary Table 3**), the final location of the sampling points was primarily determined by site accessibility. We therefore implemented a stratified sampling with targeted site selection according to severity classes (Crotteau et al., 2013), following specific rules such as maintaining a minimum distance of 50 meters between plots.

Plots consisted of a circular area of 113 m² (6 m radius), subdivided into four quadrants for the assessment of trees with a diameter at breast height (DBH) greater than 5 cm. Additionally, eight subplots of a circular area of 1.13 m² (0.6 m radius), two per quadrants were established to evaluate tree regeneration. The regeneration was categorized by height class: less than 50 cm (RH1), 50–200 cm (RH2), and greater than 200 cm (RH3).

Within each plot, the abundance and diameter at breast height (DBH) of native and exotic tree species were recorded, along with the vital status of individual trees (alive or dead), their origin (seedling or resprout), and the number of basal resprouts. The resprouts were categorized by height class: less than 50 cm (RH1), 50–200 cm (RH2), and greater than 200 cm (RH3). Regarding the assessment of tree vitality, individuals classified as ‘alive’ exhibited a vigorous main aerial structure (trunk, branches, and canopy) that was minimally affected by fire. In contrast, ‘dead’ individuals were those whose main aerial structure, including the stem, branches, and canopy, had been burned. Trees with burned crowns but showing vegetative regrowth at the base of the trunk were also categorized as ‘dead’.

### 2.4 Sampling plots distribution per severity classes

The 18 sampling plots were selected to analyze fire severity. Each plot was georeferenced with GPS coordinates and a 10 meter buffer was applied around each plot to include the GPS margin of error (3–5 meters).The median value of Sentinel pixels were extracted for each site within the buffer area. This resulted in 18 values analyzed (2–3 pixels per plot). Due to limitations in terms of access and resources, the original sampling plot design was kept after the severity classes were reclassified. However, the “severity class” was modified based on the reclassification values.

To ensure the defined severity classes constituted statistically robust and representative groups for sampling, a one-way analysis of variance (ANOVA) was performed to determine if there were significant differences in dNBR values among the three classes. A Tukey’s *post-hoc* test (HSD) was then applied at a 95% confidence level to identify which specific severity classes differed from each other.

Additionally, a spatial segregation and autocorrelation test was performed. The Multiclass Join-Count Test was used to validate the geographic configuration of the strata. This test evaluated the geographic interaction between severity classes and coordinates. To test for spatial segregation and ensure that the severity classes represented discrete territorial units and not a random or mixed distribution across the landscape, the Z-score for the joins between different severity classes was analyzed.

### 2.5 Analysis of post-fire vegetation recovery using PlanetScope images

PlanetScope images were preprocessed to cover a post-fire observation period of six years, from September 2016 to March 2023. We used 79 monthly images with less than 10% cloud cover to detect changes in vegetation after the fire. The images were obtained via the Planet Explore platform (educational license) and subjected to atmospheric correction using regressions (Equation 1). Surface reflectance was calculated from reflectance at the top of the atmosphere (TOA) using coefficients from the Planet Radiance product. This allowed atmospheric effects to be corrected for, considering molecular composition, altitude, and aerosol content (Planet, 2022). Radiance values were converted to reflectance using Equation 1. This procedure was implemented in R software using the raster package to handle raster files and the xml2 package (Wickham et al., 2025) to process metadata.

(1) $$R_{i}=DN_{i}\times C_{i}$$

Where R is the reflectance of the i band, DN is the digital level of the i band, and C is the reflectance coefficient of the i band.

Subsequently, spectral indices, including the normalized difference vegetation index (NDVI) and the modified soil-adjusted vegetation index (MSAVI2), were used to evaluate vegetation vigor (**Table 2**).

**Table 2:** Spectral indices and calculation formulas from PlanetScope bands.

Spectral index	Equation
NDVI	$\frac{IR-RED}{IR+RED}$
MSAVI2	$\;\frac{2\cdot IR+1-\sqrt{{\left(2\cdot IR+1\right)}^{2}-8\cdot (IR-RED)}}{2}$

The 18 sampling plots were selected to analyzed vegetation cover with different severity classes. Each plot was georeferenced with 10-meter buffer was applied around each plot to include the GPS margin of error (3–5 meters), which was calculated based on a 6-meter radius around the plot area. For each site, the median value of the PlanetScope pixels within the buffer area was extracted for each date in the time series. This resulted in 18 values analyzed per index per month.

We analyzed changes in vegetation photosynthetic vigor after the fire using three linear mixed models as a function of time and fire severity (Leal-Medina et al., 2024). Each model used time series data from the NDVI and MSAVI2 indices (representing vegetation vigor) as the dependent variable and post-fire time (up to six years), fire severity and their interaction as fixed factors (Equation 2). The models were implemented in the R software using the nlme package (Pinheiro et al., 2025) and adjusted means were obtained using lsmeans (Lenth, 2016). Statistical differences among groups were evaluated using multicomp (Hothorn et al., 2008).

(2) $$\gamma_{ijk}=\mu +S_{i}+T_{j}+{\left(ST\right)}_{ij}+P_{k}+e_{ijk}$$

Where *γ_ijk_* represents the response variable (vigor), *µ* corresponds to mean of vigor index, *S_i_* to the effect of fire severity, *T_j_* to the effect of post-fire time, (*ST*)*_ij_* to the severity–time interaction, *P_k_* to the random effect of plot, and *e_ijk_* to the residual error of the model.

To assess which main effects and interactions among predictors were statistically significant, a Wald test was carried out using the car package. Subsequent to the indication of significant differences by the Wald test, the Tukey’s *post-hoc* test was implemented for the purpose of identifying which post-fire periods differed significantly from each other.

Additionally, to evaluate post-fire vegetation recovery changes, we compared NDVI and MSAVI2 values between winter and summer conditions. Representative months characterized by extreme seasonal climatic conditions were selected to capture canopy dynamics during the two phenological phases of deciduous vegetation: the leaf-off period (winter) and the leaf-on period (summer). These indices were analyzed annually for a period of five years following the fire, allowing the assessment of canopy development and vegetation regeneration over time.

The independence of the model residuals was evaluated to ensure the validity of the inferences derived from the mixed-effects model. Given that the NDVI data consists of repeated measures over the same sampling units (ID) over time, identifying potential serial autocorrelation was a priority. Initially, a Durbin-Watson test (Durbin and Watson, 1950) was conducted on the normalized residuals to identify first-order autocorrelation. This evaluation furnishes a numerical metric indicative of the linear relationship, if any, between errors from successive time points. Subsequently, a Total Autocorrelation Function (ACF) plot was generated to inspect the residual dependence across extended time series (Ramsey, 1974). This dual approach, integrating a formal statistical test with a visual diagnostic, facilitates the identification of both immediate serial dependence and long-term patterns that may persist despite the fixed effects of the model.

### 2.6 Analysis of post-fire regeneration of *N. alessandrii* and invasion of *P. radiata*

The resprouting capacity of post-fire species was quantified as the proportion of individuals with basal resprouts relative to the total number of trees in each plot (Equation 3).

(3) $$\text{Resprouting Capacity by Species }(\%)=\frac{\text{Number of Individuals with Basal Resprouts}}{\text{Total Number of Individuals}}\times 100$$

Resprout density was estimated according to the previously defined height classes and for each fire-severity category. These evaluations were complemented with analysis of variance (ANOVA) followed by Tukey’s *post-hoc* tests in R using the tukeyC package (Faria et al., 2025), to identify statistically distinct groups among severity classes.

The degree of *P. radiata* invasion in the sampling plots was determined from its relative abundance, based on counts of both saplings and tree regeneration within the subplots. Seedling density (number of individuals per hectare) was calculated for each plot and fire-severity class, allowing the identification of differences among high, medium, and low-fire severity. Additionally, a comparative analysis of relative abundance across severity classes was conducted using Tukey’s *post-hoc* test in R with the tukeyC package (Faria et al., 2025).

### 2.7 Analysis of radial growth of *N. alessandrii* and *P. radiata*

To determine tree ring radial growth, in each fire severity site tree cores were sampled from ten *P. radiata* saplings and ten *N. alessandrii* resprouts per plot, with a DBH of less than 10 cm for both species. Tree cores were then dried and sanded using different grades of sandpaper (from 100 to 800 grit) to make the 28 growth rings visible for subsequent measurement and analysis.

Samples were then scanned in a HP-scanner at 3200 dpi resolution. The scanned samples were dated using the CDendro software to determine the age of the samples and subsequently analyzed in Coorecorder to determine the annual growth rate (2017 to 2021) and the cumulative growth for this period. To evaluate differences in cumulative radial growth according to fire severity and species, an ANOVA-based statistical analysis was performed followed by Tukey’s *post-hoc* tests using the tukeyC package (Faria et al., 2025). If assumptions were not met, the non-parametric Kruskal–Wallis test was applied together with Wilcoxon tests using R-Project software.

## 3 Results

### 3.1 Fire severity of the megafire in relict forests of *N. alessandrii*

The severity index (dNBR) had a mean value of 0.58, ranging from a minimum of 0.07 to a maximum of 1.35 among the three *N. alessandrii* populations affected by the ‘Las Máquinas’ megafire (**Supplementary Figure 4**). Reclassification of fire severity five years after the fire identified six sites with high severity, seven with medium severity, and five with low severity (**Supplementary Table 4**). The ANOVA analysis confirmed that the severity classes were numerically distinct and that there was no significant overlap between their means (p *<* 0.001). Tukey’s multiple comparisons test revealed that all severity classes pairs were significantly distinct from each other (p *<* 0.001 in all cases). The largest mean difference was observed between the high and low classes (difference = 0.825, *p <* 0.001). The moderate class positioned itself as a well-defined intermediate class, distancing itself from the low (difference = 0.380, *p <* 0.001) and high (difference = 0.445, *p <* 0.001) severity classes.

Generally, the reclassification values agreed with *in-situ* validation, except for the low severity site (LS-1), which was reclassified as moderate severity (MS). The MS-2, MS-4, LS-2, and LS-5 sites presented mixed pixels within different severity classes, but the median values correctly corresponded with the representative severity classes (**Supplementary Figure 6**).

The spatial analysis was using the multiclass Join-Count test confirmed a highly segregated patch structure (J(total) Z = -3.40, p *<* 0.01). There was significant positive autocorrelation within the extreme classes, with greater clustering in the high (High: High, Z = 2.66) and low (Low: Low, Z = 2.18) severity classes. Importantly, the results revealed absolute spatial segregation between the extremes of the gradient, with a total absence of direct contact between the high and low severity classes (Z = -3.48). In contrast, preferential spatial association was identified between the high and moderate severity classes (Z = 2.45), which validates a gradual transition model of fire severity rather than random class distribution.

### 3.2 Evolution of the vegetation cover in post-fire *N. alessandrii* forests using PlanetScope images

Temporal analysis of PlanetScope images revealed the rapid recovery of vegetation cover and vigor in three *N. alessandrii* remnant stands five years after the fire, compared to pre-fire data (from September 2016 to January 2017). Vigor values, represented by the Normalized Difference Vegetation Index (NDVI) and the Modified Soil-Adjusted Vegetation Index (MSAVI), showed a clear and strong positive correlation between both indices (r = 0.99).

The residuals analysis confirmed that the model effectively accounted for the primary temporal dynamics of the data. The Durbin-Watson statistic was calculated at 1.86, which falls within the optimal range (near 2.0), indicating that there is no significant first order serial autocorrelation.

**Figure 2:**
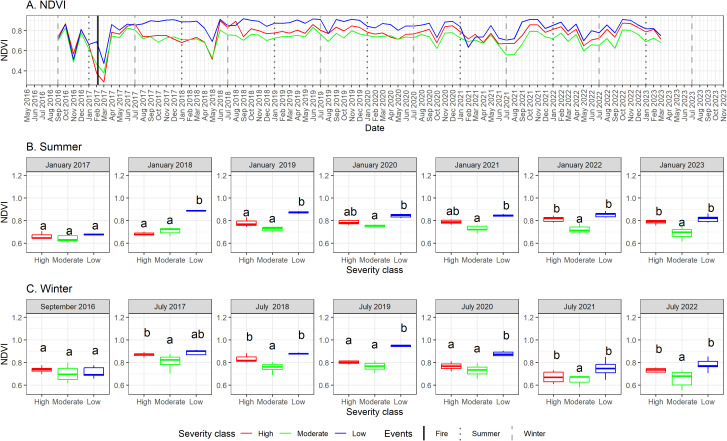
**(A)** Temporal analysis of vegetation cover using NDVI for sites affected by the low, medium and high fire severity values since September 2016 up to March 2023; **(B)** NDVI values during different post-fire summer seasons (January); **(C)** NDVI values during different post-fire winter seasons (July).

The autocorrelation function (ACF) analysis of the residuals (**Supplementary Figure 7**) confirmed the validity of the statistical model by showing satisfactory independence in the immediate lags. A significant cyclical pattern with peaks in months 18 and 36 was identified, revealing a periodicity of approximately one and a half years in vegetation dynamics. This behavior is consistent with the NDVI time series (**Figure 2A**).

During the first year after the fire (2017–2018), the time series revealed clear differences in vegetation vigor, as indicated by NDVI and MSAVI2, between sites affected by moderate–high and low fire severity (**Figure 2A**). However, toward the end of the time series, sites impacted by high fire severity converged toward vigor values comparable to those observed in low-severity sites (**Figure 2B**), indicating a marked recovery over time.

In addition, significant variations in vegetation vigor were observed when comparing summer and winter periods following the fire. During the first post-fire winter (July 2017), sites classified as high and moderate severity exhibited high NDVI values, similar to those of low-severity sites (approximately 0.9). In contrast, during the following winter, a decrease in vegetation vigor was observed in sites affected by high and moderate severity (**Figure 2C**). During the summer months, low-severity sites maintained high vigor values, ranging from 0.8 to 0.9, whereas sites affected by high and moderate severity showed lower vigor compared to the first winter after the fire (July 2017 to January 2018), followed by a gradual increase over time (**Figure 2B**). Vegetation vigor is a key indicator of post-fire vegetation cover establishment, with values between 0.6 and 0.9 corresponding to dense vegetation with high photosynthetic activity.

Sites affected by low fire severity had high NDVI values compared to sites affected by moderate or high fire severity. This pattern was maintained during the first two years after the fire. Five years after the fire, extensive *Pinus radiata* invasion in sites with high fire severity, combined with the absence of *Nothofagus alessandrii* trees, likely led to increased NDVI values similar to those in sites with low fire intensity. Moderately affected sites presented mixed patterns and different vigor values of fire severity where regenerated native tree cover and invasive species co-occurred in similar proportions.

### 3.3 Changes in the composition and structure of post-fire *N. alessandrii* forests

Field data revealed significant changes in the composition and structure of the post-fire vegetation associated mainly with the recruitment of *P. radiata* and *N. alessandrii* species.

#### 3.3.1 *P. radiata* invasion

We reported a direct relationship between fire severity and degree of *P. radiata* invasion five years after the event. In sites affected with high and moderate fire severity, the mean density of *P. radiata* reached 9,760 and 9,063 individuals per hectares, respectively. In the case of sites affected low severity sites the average density of *P. radiata* was lower with 1,657 individuals per hectare (**Table 3**).

**Table 3:** Regeneration density of *P. radiata* seedlings (individuals per hectare) invading *N. alessandrii* forests burned with different fire severity.

Severity	Number of plots	Mean	Min	Max	SD
High	6	9,762.9^a^	4,420.9	18,789.1	4,913.8
Moderate	7	9,062.9^a^	1,105.2	19,894.3	7,389.4
Low	5	1,657.8^b^	1,105.2	2,210.4	781.5

#### 3.3.2 Early post-fire regeneration response of *N. alessandrii* forest

**Figure 3:**
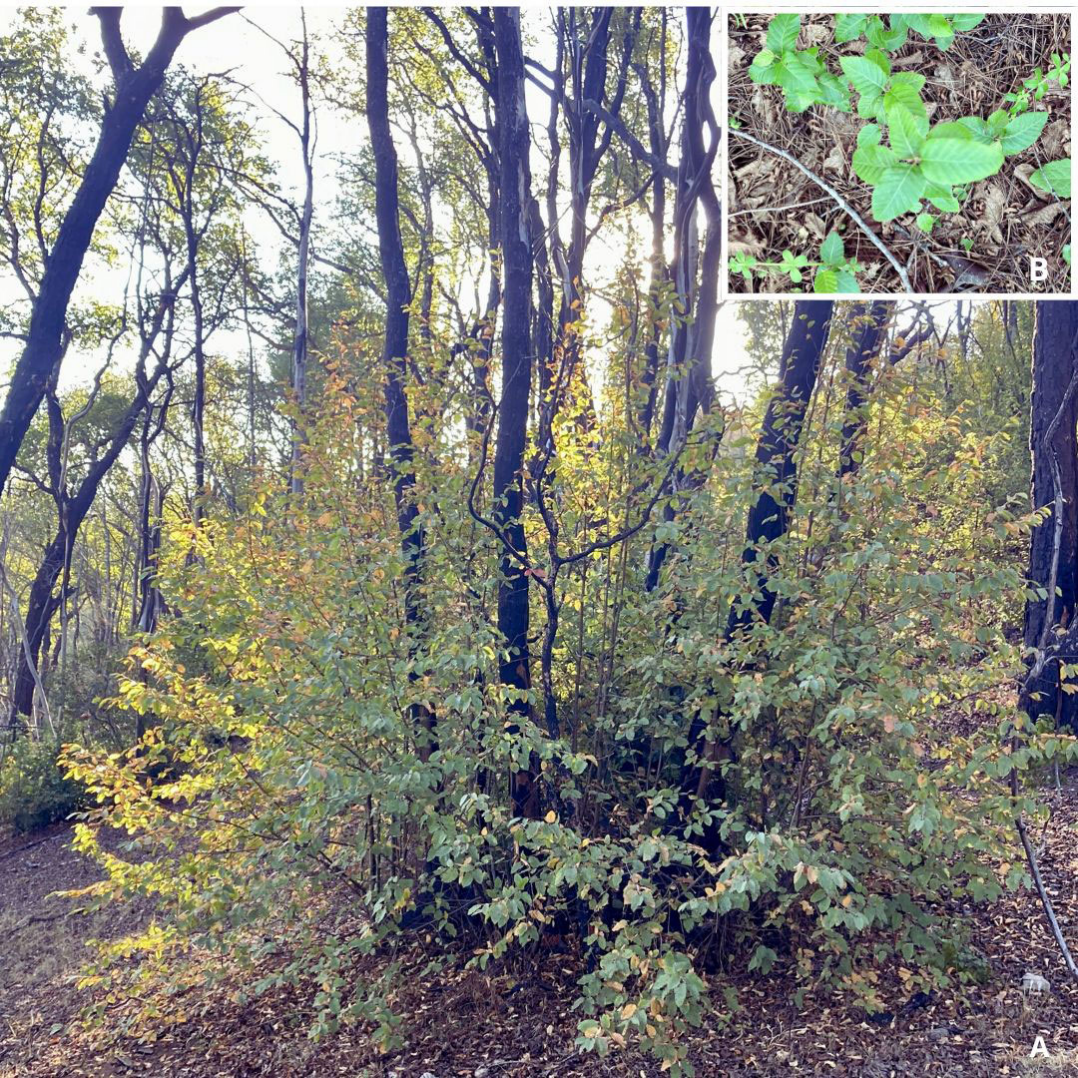
**(A)** Resprouting plants and **(B)** Post-fire seedling recruitment in *N. alessandrii* forests.

Five years after the fire, *N. alessandrii* forest showed both vegetative and seedling recruitment (**Figures 3A, B**). Vegetative recruitment was the most frequently observed strategy for *N. alessandrii*. In areas affected by moderate and high fire severity, this species reported over 70% of basal resprouting. In contrast, in low severity sites the resprouting capacity of *N. alessandrii* trees was less than 50%. Other species, such *Cryptocarya alba* and *N. glauca* also exhibited high resprouting and crown regrowth capacities in the sampled sites, particularly *C. alba*, which reached over 90% resprouting in high severity sites (**Table 4**).

**Table 4:** Post-fire resprouting capacity of *N. alessandrii* and other native species in sites affected by low, moderate and high fire severity.

Severity class	Species	Density (Ind/ha)	Min	Max	Resprouting capacity (%)
High	*N. alessandrii*	973	354	1768	72%
	*C. alba*	752	354	2299	91%
	Others	15	0	88	100%
Moderate	*N. alessandrii*	884	88	2653	79%
	*C. alba*	88	177	354	64%
	*N. glauca*	51	0	88	17%
	Others	63	88	265	83%
	*N. alessandrii*	513	88	1061	48%
Low	*C. alba*	637	265	973	75%
	Others	230	88	442	76%

*The density (ind/ha) corresponds to the individuals with the presence of vegetative regrowth in plots of 113 *m*^2^. Other species include the following: *Quillaja saponaria*, *Lithraea caustica*, *Luma chequen*, *Peumus boldus*, *Luma apiculata*, *Persea lingue*, Kageneckia oblonga, *Azara integrifolia* and *Citronella mucronata.*

**Figure 4:**
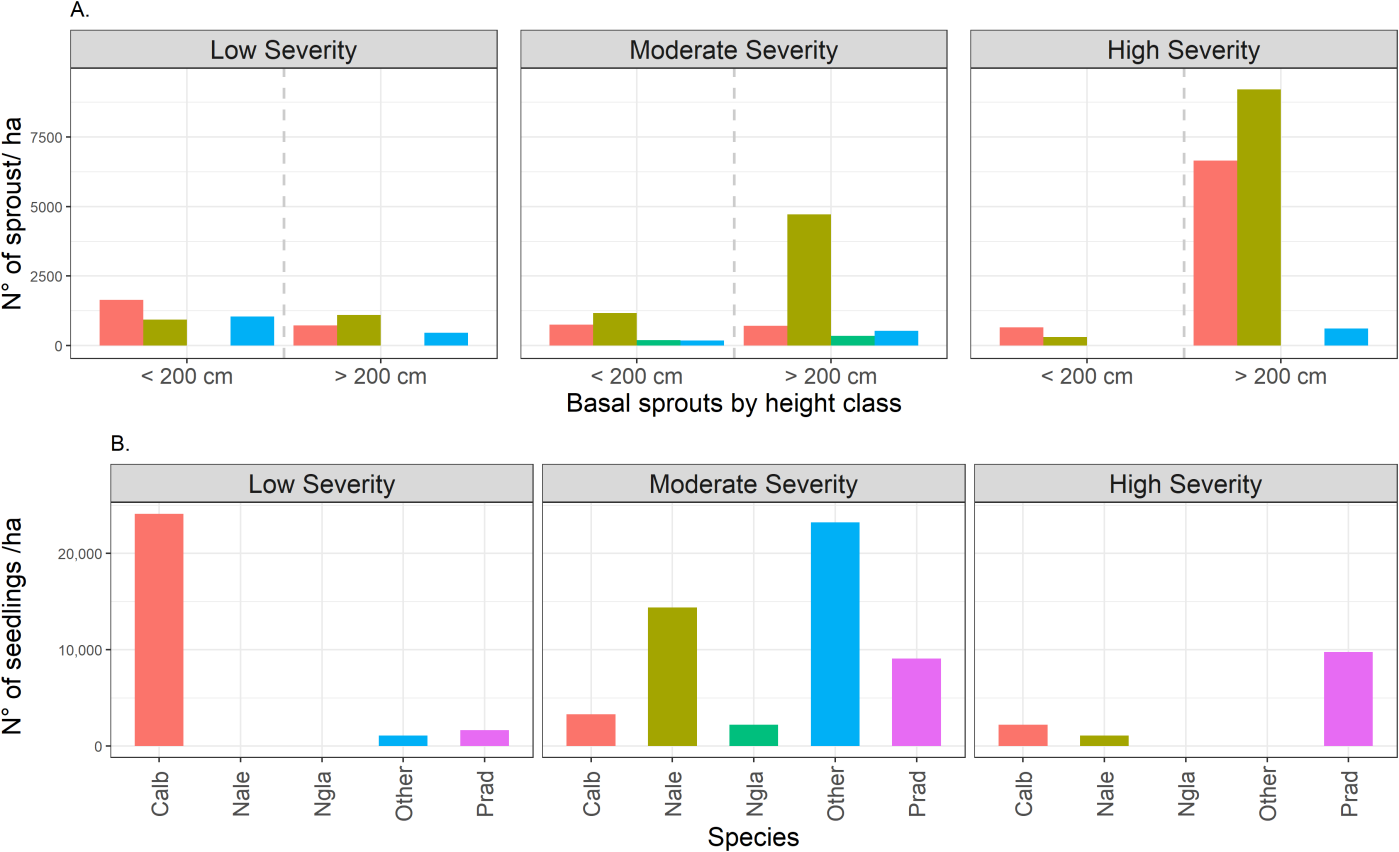
**(A)** Sprout density according to the height classes of resprouting plants for each fire severity class after five years of the fire event: Resprouting plants (*<* 200 cm) and Sampling (*>* 200 cm); **(B)** Density of seedlings (*<* 200 cm) of main tree species recruiting after different fire severity. Calb, *C. alba*; Nale, *N. alessandrii*; Ngla, *N. glauca*; Prad, *P. radiate*; Other, Other tree species.

Sprout density varied significantly between severity classes (**Figure 4A**). Low-severity sites exhibited relatively low densities of resprouting plants (RH1 and RH2) and saplings (RH3). High-severity sites exhibited the highest overall densities, especially of saplings.

In addition, *C. alba* recruited densely via seeds, especially in sites with low severity, reaching densities exceeding 20,000 seedlings per hectare (**Figure 4B**). At moderate-severity sites, *Nothofagus alessandrii* and other native species exhibited elevated seedling densities, while *P. radiata* was also abundant under both, moderate and high fire severity. Overall, seedling recruitment was observed in all disturbance categories, though there was significant variation in species composition and density among severity classes.

### 3.4 Post-fire radial growth of Pinus radiata and Nothofagus alessandrii

**Figure 5:**
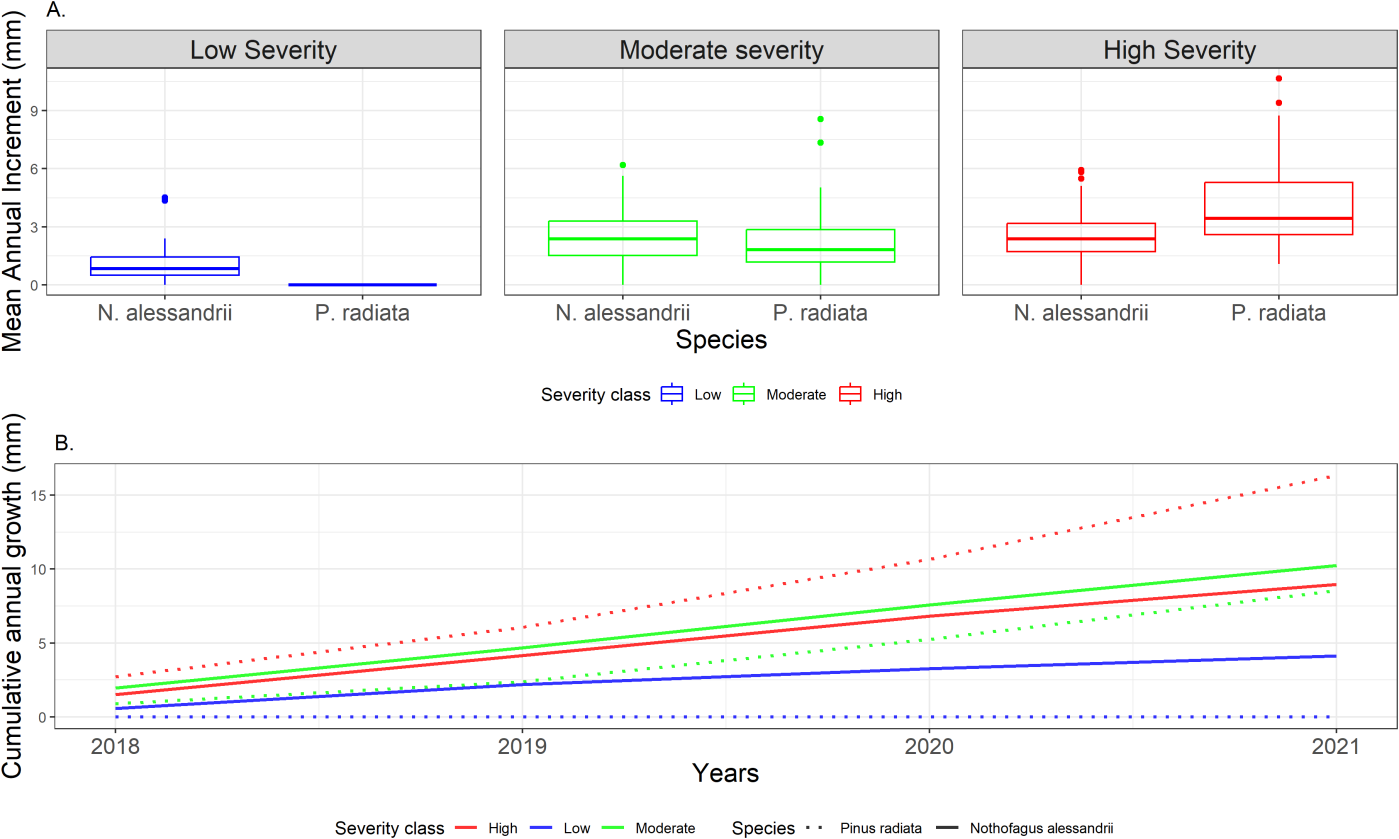
**(A)** Mean annual increment (MAI) per species and severity classes; **(B)** Cumulative annual growth of *Nothofagus alessandrii* (dashed line) and *Pinus radiata* (solid line) under different fire severity classes.

Mean annual increment (MAI) of tree rings revealed significant differences between *P. radiata* and *N. alessandrii* under different fire severity classes. In sites affected by high fire severity, *P. radiata* exhibited an average radial growth rate of 4.08 mm per year compared to the average radial growth rate of 2.53 mm per year of *N. alessandrii* resprouts (*P <* 0.05). In sites affected by moderate fire severity, *N. alessandrii* showed a similar average radial growth rate (2.55 mm per year) than the radial growth found under high fire severity. Furthermore, compared to *P. radiata*, which grew at an average rate of 2.15 mm per year not significative differences were observed (*P >* 0.05). In sites affected with low fire severity, only *N. alessandrii* individuals were recorded, with an average radial growth of 1.03 mm per year (**Figure 5A**).

In sites affected by high severity (red) *P. radiata* exhibited the greatest cumulative growth, whereas this decreased in sites of moderate (green) and low (blue) severity. For *N. alessandrii*, the effect of severity did not show clear differences in cumulative growth between sites with moderate and high severity, although it differs from sites with low severity (**Figure 5B**).

## 4 Discussion

### 4.1 Fire severity assessment and complementary approaches

The results obtained using the dNBR index are consistent with the severity classes determined in previous studies of *N. alessandrii* forests after ‘Las Máquinas’ megafire (Valencia et al., 2018; Santelices-Moya et al., 2022). However, when analyzing the severity of each plot and buffer area (**Supplementary Figures 5**, **S6**), a mixture of values associated with different severity categories was evident in some cases. This could indicate that the location of the plots could be associated with transition zones. Nevertheless, the exclusive use of spectral indices has limitations, particularly in representing the lower canopy strata in areas of sparse vegetation (Hudak et al., 2007; Fassnacht et al., 2021). After fires, vegetation change and biological legacies (i.e., trees and other surviving understory species) promote heterogeneity in forest cover, which can influence the interpretation of the severity indices (Miller and Thode, 2007; Soverel et al., 2010; Pérez et al., 2017). Additionally, the presence of dead trees in a post-fire forest landscape may notably influence the dNBR spectral signal. Standing snags may shade a significant proportion of the ground, which can affect passive optical satellite signals and consequently impact the dNBR values (Fassnacht et al., 2021).

As spectral indices capture the magnitude and immediate impact, they do not always accurately reflect the true effects of fires on forest structure or differentiate between different fire severity classes (Morgan et al., 2014). The delayed impact of fire on mortality is a major source of uncertainty regarding the ecological effects of low or moderate fire severity forests (Robbins et al., 2022). Integration of satellite observations with field data, in concert with adjusted severity classes thresholds, have been demonstrated to significantly enhance the accuracy of severity assessments (Key and Benson, 2006; Santelices-Moya et al., 2022). This approach has been found to be particularly effective in areas of low or moderate fire severity, where pre-fire conditions play a crucial role in the short term.

In this regard, the dynamics of regeneration processes is largely determined by pre fire composition and structural forest attributes (Viana-Soto et al., 2020). Also, the proportion of canopy cover loss becomes a critical variable for the detection and interpretation of differential fire severity classes (Sankey et al., 2021). Integrating high-resolution UAS (drone) imagery either for visual interpretation or the derivation of structural metrics represents a promising complement to satellite-based assessments. Such integration enhances the spatial and structural precision of post-fire monitoring, refines fire severity classifications at finer scales, and provides a valuable framework for advancing future research on forest recovery dynamics, dead trees and ecosystem resilience (Fassnacht et al., 2021; Möhring, 2024).

### 4.2 Monitoring vegetation recovery after fire

Monitoring *N. alessandrii* forest recovery using PlanetScope imagery enables systematic documentation of post-fire regeneration processes at high spatial resolution (Pickell et al., 2016). Time series of spectral indices, including NDVI and the MSAVI2 provide complementary information on canopy greenness, vegetation vigor, and moisture dynamics, allowing the characterization of vegetation responses before and after fire events (Miller et al., 2019; Sturm et al., 2022; Farmonov et al., 2023). This confirms our first hypothesis, variations in canopy cover and spectral greenness alone capture only part of the recovery process and do not necessarily reflect full forest restoration, which also depends on physiological functioning (Yan et al., 2025) and the interaction of biophysical, biological, and biogeochemical processes (Liu et al., 2008).

The main tree species that established in burned areas after fire were *N. alessandrii* and *P. radiata*, with the latter predominating in sites affected by higher fire severity. *N. alessandrii* burned forest affected by high and moderate fire severity initially showed delayed recovery but reached vigor values comparable to low severity sites in five years, suggesting rapid growth of surviving trees, young trees and regeneration development of the understory (Khoury and Coomes, 2020). The species associated with post-fire regeneration in *N. alessandrii* forests may include native and exotic species (San Martín, 2022).

Regarding representative dates of seasonal, the spectral indices, NDVI and MSAVI2 captured vegetation changes primarily related to vigor (Alegria, 2022). The first acquisition date after fire corresponds to the winter season, when deciduous vegetation sheds its foliage. During this period, high and moderate severity sites exhibited elevated NDVI values (first year after fire), likely associated with the early establishment of evergreen shrubs and herbaceous species. This phenomenon could be linked to the germination seed bank stimulated by the first autumn or winter rains. Additionally, a dense recruitment of *P. radiata* could have also contributed to high NDVI values, considering its ability to establish in *N. alessandrii* forest gaps often involving the exotic species (San Martín, 2022).

During summer, both indices NDVI and MSAVI2 showed an increase in vegetation vigor up to one year after the fire at low severity sites. These findings are not indicative of water stress processes, and it is directly related to the spring growth of deciduous vegetation typical of these forests. In contrast, sites affected by high and moderate fire severity reached similar values of vigor to those of low-severity sites two years after fire, reflecting the relatively fast regeneration process after fire (San Martín, 2022; González et al., 2022a).

Overall, these results confirm that spectral indices are valuable tools for monitoring post-fire recovery (Section 4.3). Nevertheless, their interpretation should account for the severity of fire, seasonality, and structural composition of vegetation, and be complemented with field data to accurately assess ecosystem composition and functionality (Epting and Verbyla, 2005).

### 4.3 Post-fire regeneration dynamics and the impact of *Pinus radiata* invasion on relict *Nothofagus alessandrii* forests

*Pinus radiata* invasion within *N. alessandrii* relict forests has triggered profound structural and functional transformations with detrimental ecological consequences (Catford et al., 2012). *Pinus radiata* invasion has also been reported in different studies (Bustamante and Castor, 1998; Bustamante and Simonetti, 2005; Gómez et al., 2019), especially after megafires events that represent a rising ecological pressure (González et al., 2022a; Leal-Medina et al., 2024). This pattern is largely explained by the pre-fire forest structure, in which large *P. radiata* trees were already invading the native *N. alessandrii* forest due to the large surrounding forest plantation matrix (Bustamante and Simonetti, 2005; González et al., 2022a). The megafire, triggered the release of a massive amount of seeds, which rapidly germinated and established resulting in a high density of seedlings, especially in areas affected by high fire severity (González et al., 2022a, b). Although the density of *P. radiata* recorded in high severity sites was high (9,762 seedlings/ha compared to 1,250-1,500 seedlings/ha planted for forest companies), it was lower than in other neighboring studies (Leal-Medina et al., 2024).

Severely burned sites provided favorable regeneration niches with favorable conditions for *P. radiata* invasion due to poor forest coverage, reduced competition and high radiation (Richardson, 2011). All these factors led to the widespread dominance of *P. radiata* seedlings, which has homogenized the landscape and created a highly competitive environment for native species (Guo et al., 2015; Pauchard et al., 2006). In south-central Chile and north Patagonia in Argentina, several studies have documented a similar invasion process to native forests by pine species (Peña et al., 2008; Langdon et al., 2010). For example, *Pinus contorta* has exerted a negative effect on the diversity of vascular plants in natural habitats. These species have a rapid growth, reducing the impact richness and cover of native species as they expand their invasive pine canopies (Taylor et al., 2016; Franzese et al., 2017).

Moreover, at high-severity sites, the main forest cover was burned and the area was heavily invaded by *P. radiata* five years after the fire, resulting in almost non-existent *N. alessandrii* seedlings due to the absence of seed sources. Additionally, post-fire conditions such as soil heating and increased solar radiation at the edges of burned patches can lead to seedling dehydration and mortality, while dense canopy cover from invasive pine seedlings may further suppress natural regeneration of native species (San Martín, 2022).

In contrast, areas affected by low fire severity maintained a forest structure more similar to pre-fire conditions, which limited pine invasion and promoted the formation of shaded microhabitats favorable for the germination and establishment of *N. alessandrii* and other native species. This underscores the importance of canopy persistence in facilitating post-fire recovery (San Martín, 2022). A particular scenario was observed in areas of moderate fire severity, where germination rates of *N. alessandrii* seeds have been reported to reach approximately 15,000 seedlings per hectare (Section 3.2.2). Although the vegetation cover remained low, these conditions seem ideal for the germination of native species, such as *C. alba* and *N. glauca*. However, such areas are highly susceptible to physical damage caused by fallen trees and to subsequent pine invasion. The seed germination mechanism of *N. alessandrii* plays a crucial role in promoting seed dispersal and maintaining the genetic diversity of new generations, thus supporting the conservation and adaptive capacity of these endangered populations (San Martín, 2022; Santelices-Moya et al., 2023). Therefore, the presence of adult, seed-producing individuals is essential to ensure natural regeneration and effective seed dispersal (San Martín, 2022).

In MCF where Mediterranean vegetation ecosystems are prevalent, the most common mechanism of recruitment for many native species is the ability to sprout after a fire (Montenegro et al., 2004; Smith-Ramírez et al., 2022). However, this capacity is conceived not as an adaptation to recurrent fire regimes but rather an evolutionary vestige (Pausas, 2012). In our study area, high-severity affected sites presented the highest density of resprouts, primarily *N. alessandrii* (973 resprouts/ha) and *C. alba* (752 resprouts/ha). Moderate and low severity burned areas also presented a high resprout density after the fire. *N. glauca* showed low basal resprouting being the only species to show epicormic sprouts from the crown (White et al., 2020; González et al., 2022a, b).

Post-fire environmental conditions can directly influence species growth rates. Although populations often experience similar post-fire conditions in terms of soil type, temperature, and annual precipitation, other factors such as canopy cover vary substantially (Yan et al., 2025; Viana-Soto et al., 2020). These structural differences can lead to considerable variability in forest productivity, radial or height growth (Álvarez et al., 2013). Typically, after fire events, it is possible to identify a cohort of trees that established during the same post-disturbance period. Within this cohort, growth rates often differ depending on the species involved and their regeneration mechanisms, such as sprouting or seed-based establishment (González et al., 2014). In the case of *N. alessandrii* forests, fire severity directly influenced the density, height, and radial growth of sprouts. Low severity sites generally resulted in sprouts under 2 m height, with an mean annual increment (MAI) of tree rings approximately 1.03 mm per year, whereas medium and high fire severity sites showed similar radial growth (2.53–2.55 mm per year), with sprouts reaching more than 2 m five years after fire.

In the case of *P. radiata*, post-fire conditions can resemble those of a plantation because many individuals of the same age establish simultaneously, albeit with variable stand density, on sites that have suffered extensive canopy loss. The development of forest cover and subsequent canopy closure are directly linked to growth, which is one of the primary drivers of productivity in pine plantations (Barrios et al., 2017). A peak in *P. radiata* growth has been reported at around four to five years of age, with comparable growth patterns documented in New Zealand, Australia and Chile (Lasserre et al., 2009; Barrios et al., 2017; Ivković et al., 2013). The observed trend is consistent with the exponential increase in radial growth recorded between 2021 and 2022, four years after the fire. In high severity sites where post-fire canopy cover is low and stand density is high *P. radiata* can achieved growth rates of up to 4.08 mm per year, whereas under conditions of greater competition, the rate may declined to approximately 2.15 mm per year.

Despite the abundant resprouting of *N. alessandrii* in sites that were severely burned, the concurrent high density of *P. radiata* seedlings and the significantly greater radial growth of this invasive conifer (p *<* 0.05), which confirm our second and third hypotheses, place the successful regeneration of native species and particularly of *N. alessandrii* forests at a serious risk.

### 4.4 The collapse of the relict *N. alessandrii* forests

One of the most critical outcomes after the impact of high severity fires is the formation of new mixed forest stands dominated by exotic species, resulting in an irreversible degradation process and a shift toward an alternative stable state in which native species are progressively displaced. This altered forest structure increases the risk of ecosystem collapse and may accelerate the extinction of *N. alessandrii*. Moreover, the enhanced connectivity and flammability of the vegetation matrix promoted by *P. radiata* can reinforce fire recurrence through positive feedback loops, further compromising the survival of these relict ecosystems (González et al., 2020, 2021, 2022a).

Therefore, the main recommendations for ensuring the permanence of relict forests and avoid their extinction are:

- Monitoring and control of invasive species: Implement monitoring and control programs for *P. radiata* in fire-affected areas, prioritizing highly threatened ecosystems such as Maulino forests dominated by *N. alessandrii* and *N. glauca*. It is essential to establish an early detection system for biological invasions in burned areas, using remote sensing monitoring (satellite images or UAV data) or field surveys that can quantify early invasions. Regulations must be strengthened to ensure shared responsibility among forestry companies in preventing and controlling the spread of invasive species in Maulino forests and other ecologically valuable ecosystems.
- Ecological Restoration: Design and implement ecological restoration plans that include controlling invasive species and reforesting or enriching areas with native species under conservation status, such as *N. alessandrii* and *N. glauca*. Early post-fire management to control invasion involves removing young pines from areas affected by high and moderate fire severity as a high priority, within two to five years after the fire. Building long-term partnerships between public agencies, forestry companies, local communities and non-governmental organisations (NGOs) is essential for deploying effective, landscape-scale restoration strategies. These strategies should include ‘buffer’ areas between pine plantations and native forests, taking into account the dispersal patterns of *P. radiata* to prevent future invasions and reduce the spread of fires.
- Community Participation and Environmental Education: Encourage the participation of local communities in restoration programs and provide training in identifying species of conservation concern and controlling invasive species. Furthermore, fostering sustainable economic initiatives based on the conservation of the Maulino forest, such as ecotourism, could improve the protection of its endangered species.

## Data Availability

The original contributions presented in the study are included in the article/**Supplementary Material**. Further inquiries can be directed to the corresponding author.
