# Supplementary material for: Impacts of fire severity and exotic invasion of Pinus radiata on post-fire regeneration of relict Nothofagus alessandrii forests in central Chile
Source: Front Plant Sci. 2026 Apr 16;17:1772087. doi: 10.3389/fpls.2026.1772087 (PMC13155412; doi:10.3389/fpls.2026.1772087)
Supplement: Supplementary file 1 [file DataSheet1.pdf]

Supplementary Material

1 SUPPLEMENTARY DATA

2 SUPPLEMENTARY TABLES AND FIGURES

2.1 Tables

Table S1. Burn severity Classification based on dNBR values Key y Benson. (2006)

Severity Class	dNBR (values)
Not burn	$<$ - 0.1
Low	-0.1-0.1
Moderate low	0.1 - 0.27
Moderate high	0.27 - 0.44
High	$>$ 0.66

Table S2. Burn severity re-classification based on dNBR values

Severity Class	dNBR (values)
Low	$<$ 0.34
Moderate	0.35 – 0.84
High	$>$ 0.85

Table S3. Topographic characteristics in sampled plots base on Copernicus DEM is a Digital Surface Model- 30 m resolution

Site	Severidad (reclass)	Aspect (mean)	Aspect	Slope (°)
HS-1	High	193.62	S	24.48
HS-2	High	188.29	S	29.96
HS-3	High	190.22	S	29.44
HS-4	High	222.89	SW	12.66
HS-5	High	175.46	S	16.85
HS-6	High	138.56	SE	27.41
LS-1	Moderate	137.43	SE	28.99
LS-2	Low	125.30	SE	21.74
LS-3	Low	172.78	S	25.19
LS-4	Low	156.93	SE	21.06
LS-5	Low	192.37	S	26.64
LS-6	Low	161.03	S	29.84
MS-1	Moderate	114.20	SE	16.13
MS-2	Moderate	210.03	SW	18.53
MS-3	Moderate	220.99	SW	19.82
MS-4	Moderate	228.67	SW	4.49
MS-5	Moderate	258.03	W	15.31
MS-6	Moderate	216.20	SW	13.92

Table S4. Severity rating in sampled plots in *N. alessandrii* forests

ID	Validación	dNBR (value)	Severity class (Key & Benson, 2006)	Severity class (final)
HS-1	High	1.069	High	High
HS-2	High	1.024	High	High
HS-3	High	1.103	High	High
HS-4	High	0.984	High	High
HS-5	High	1.026	High	High
HS-6	High	1.075	High	High
MS-1	Moderate	0.704	High	Moderate
MS-2	Moderate	0.727	High	Moderate
MS-3	Moderate	0.509	Moderate High	Moderate
MS-4	Moderate	0.648	Moderate High	Moderate
MS-5	Moderate	0.586	Moderate High	Moderate
MS-6	Moderate	0.496	Moderate High	Moderate
LS-1	Low	0.544	Moderate High	Moderate
LS-2	Low	0.347	Moderate High	Low
LS-3	Low	0.221	Moderate Low	Low
LS-4	Low	0.171	Moderate Low	Low
LS-5	Low	0.252	Moderate High	Low
LS-6	Low	0.118	Moderate Low	Low

2.2 Figures

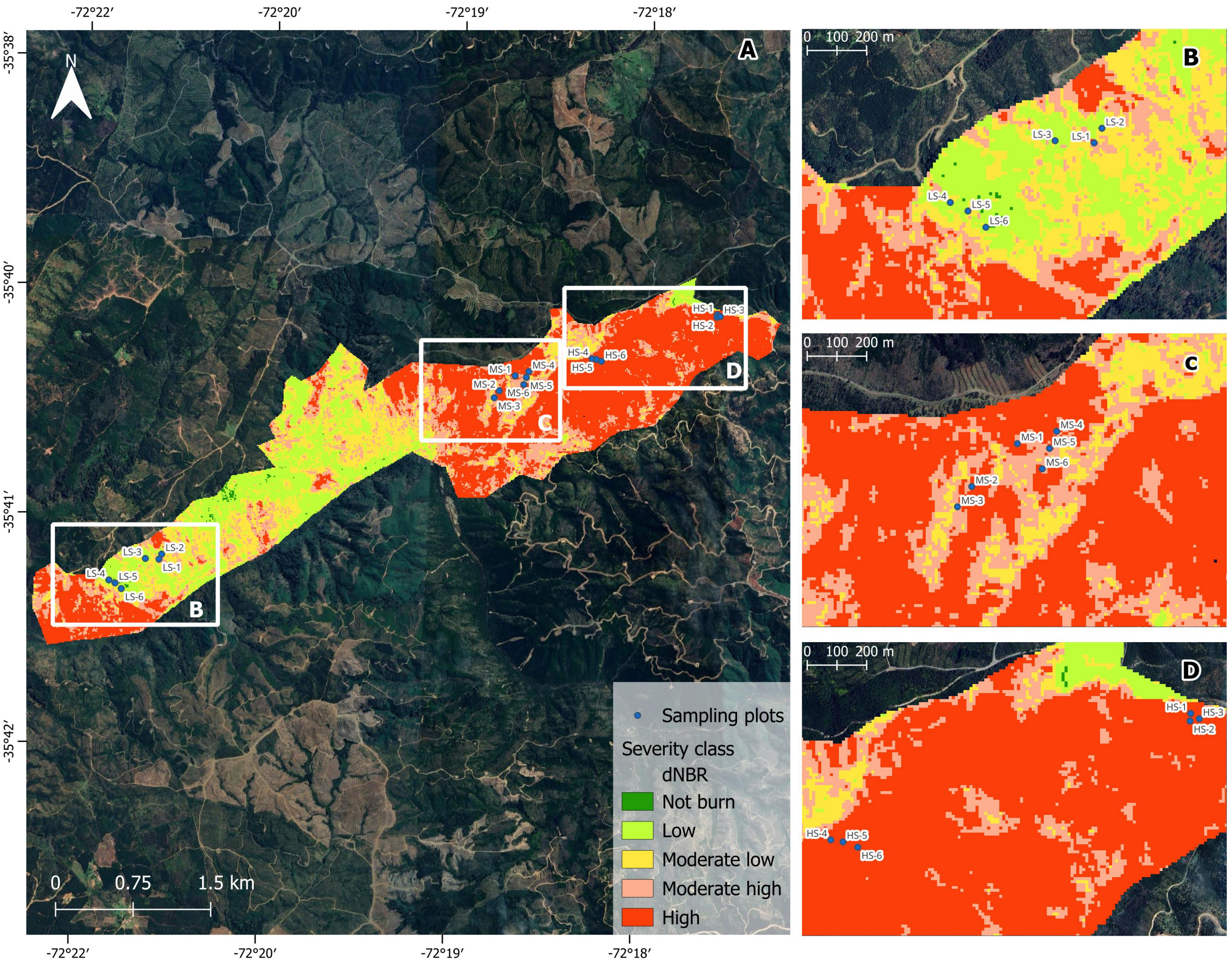

Figure S1. A. Fire severity based on the dNBR index and classes proposed by Key and Benson (2006); B) Sampling sites are affected with high severity: C) Sampling sites affected with moderate severity; D) Sampling sites affected with low severity

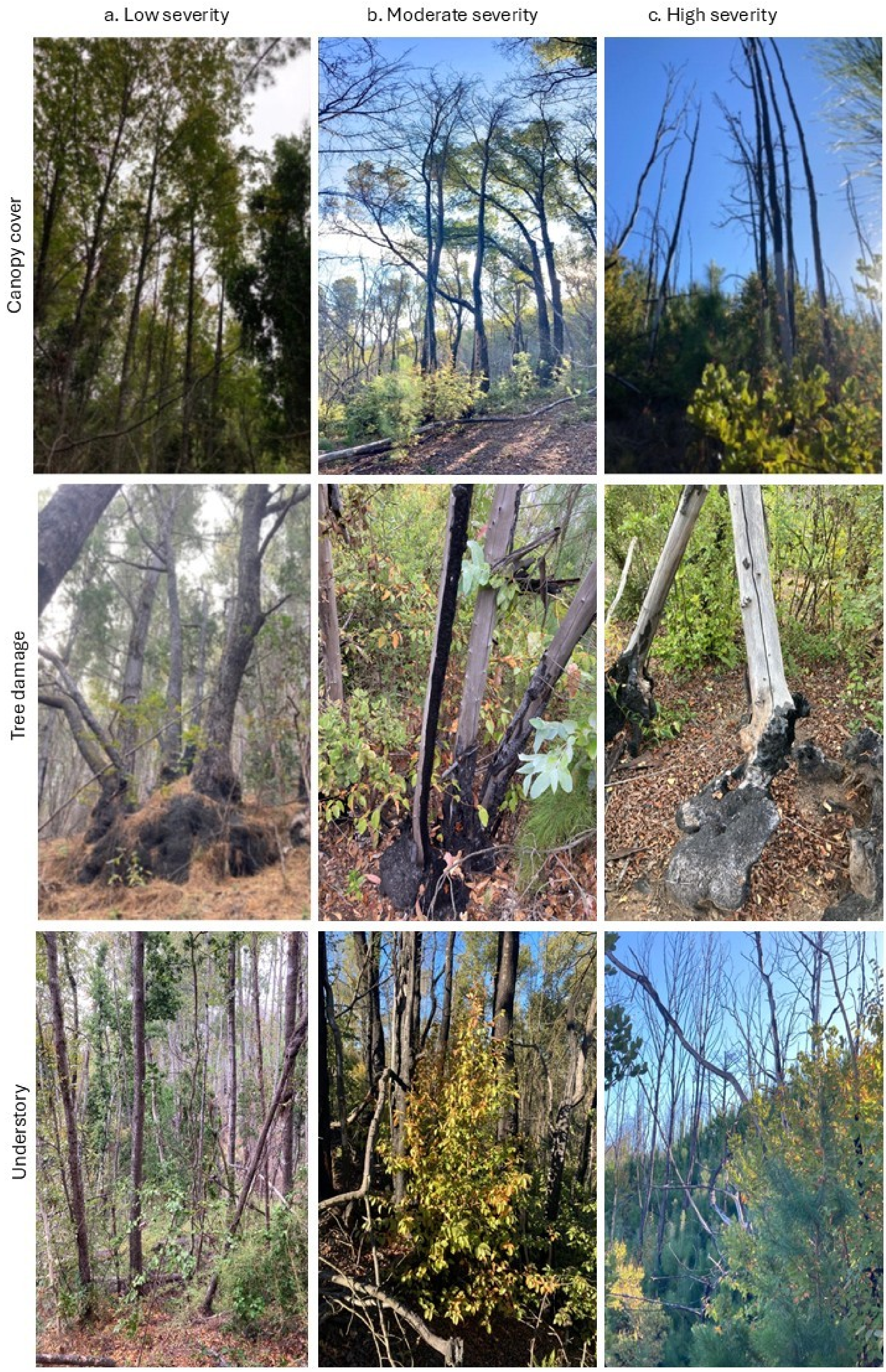

Figure S2. Severity effect per class

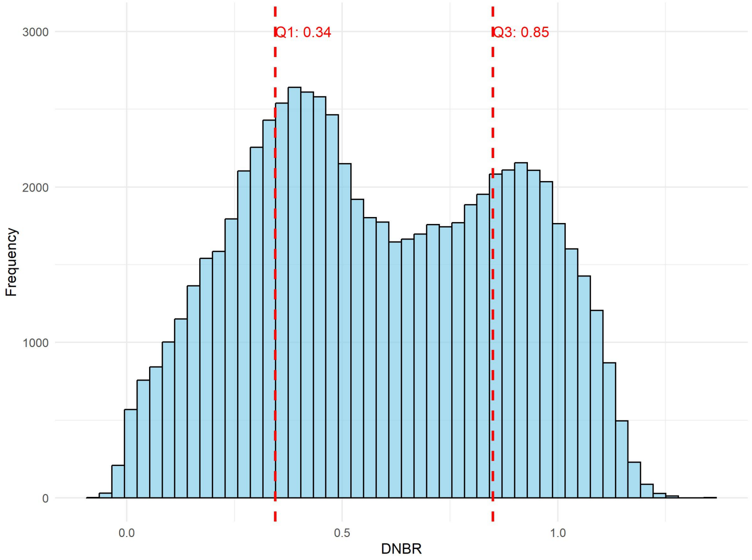

Figure S3. Histogram of DNBR Raster Values

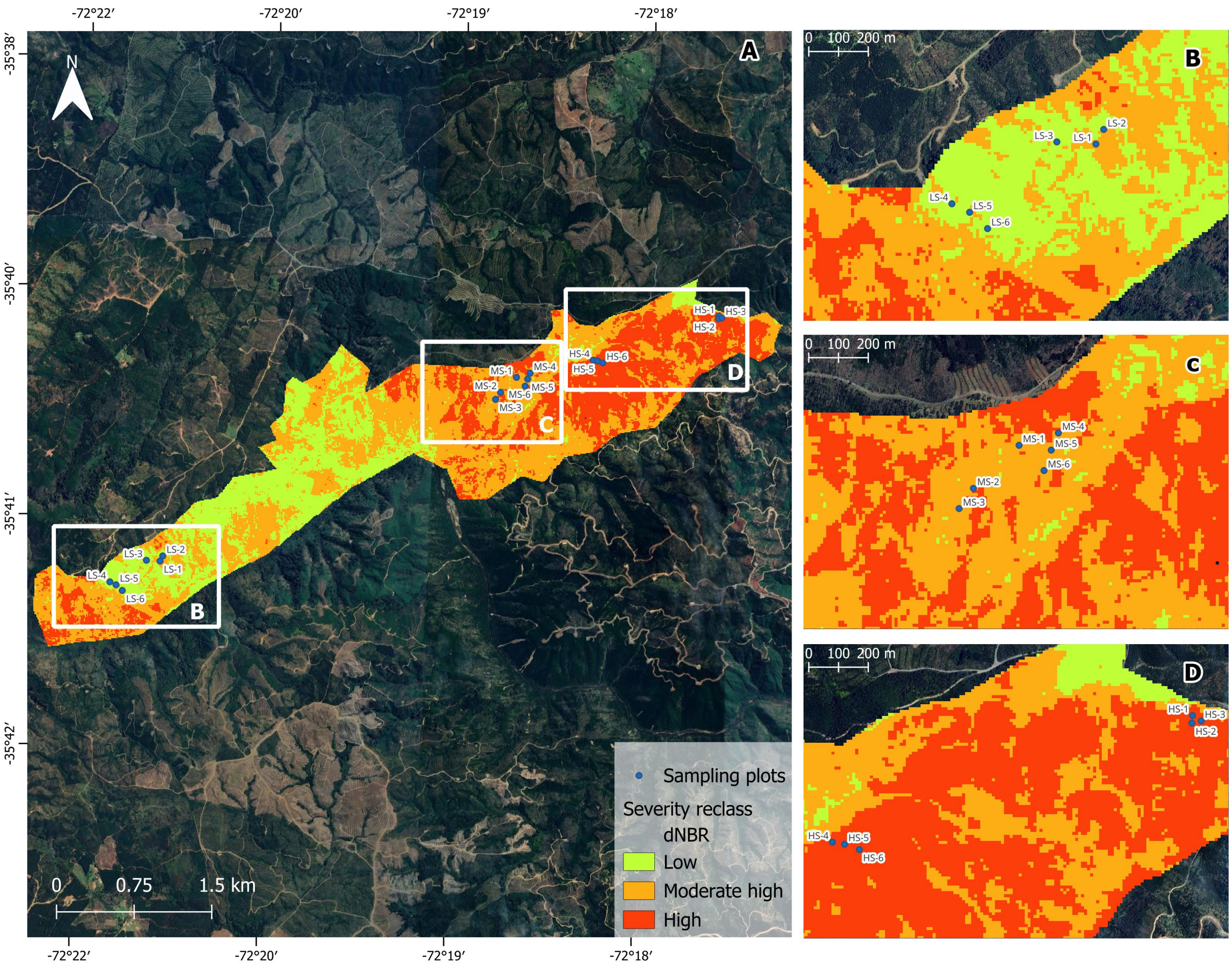

Figure S4. A. The dNBR index reclassifies fire severity based on our threshold ; B) Sampling sites are affected with high severity: C) Sampling sites affected with moderate severity; D) Sampling sites affected with low severity

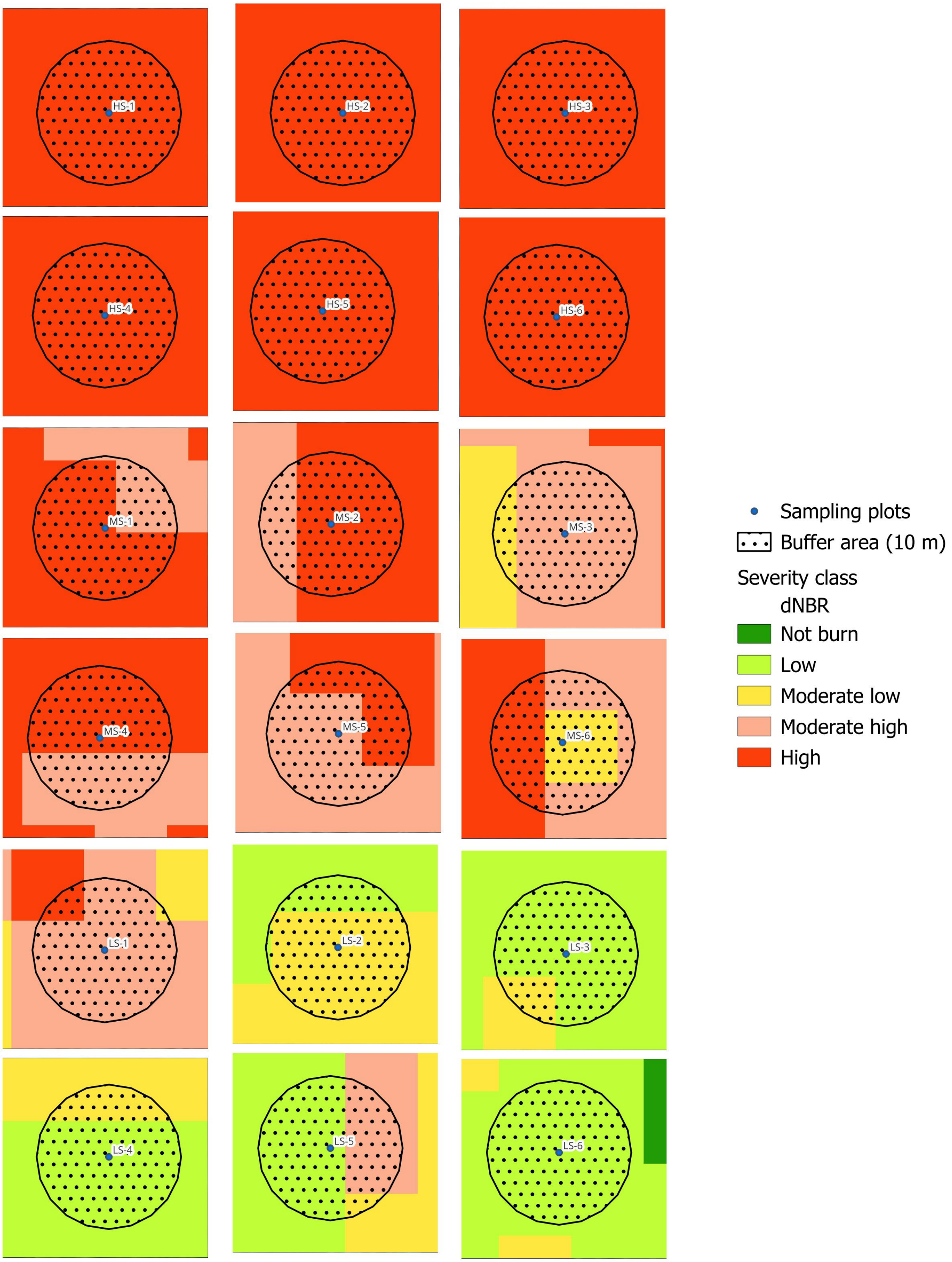

Figure S5. Sampling sites on dNBR index and the classes proposed by Key and Benson (2006)

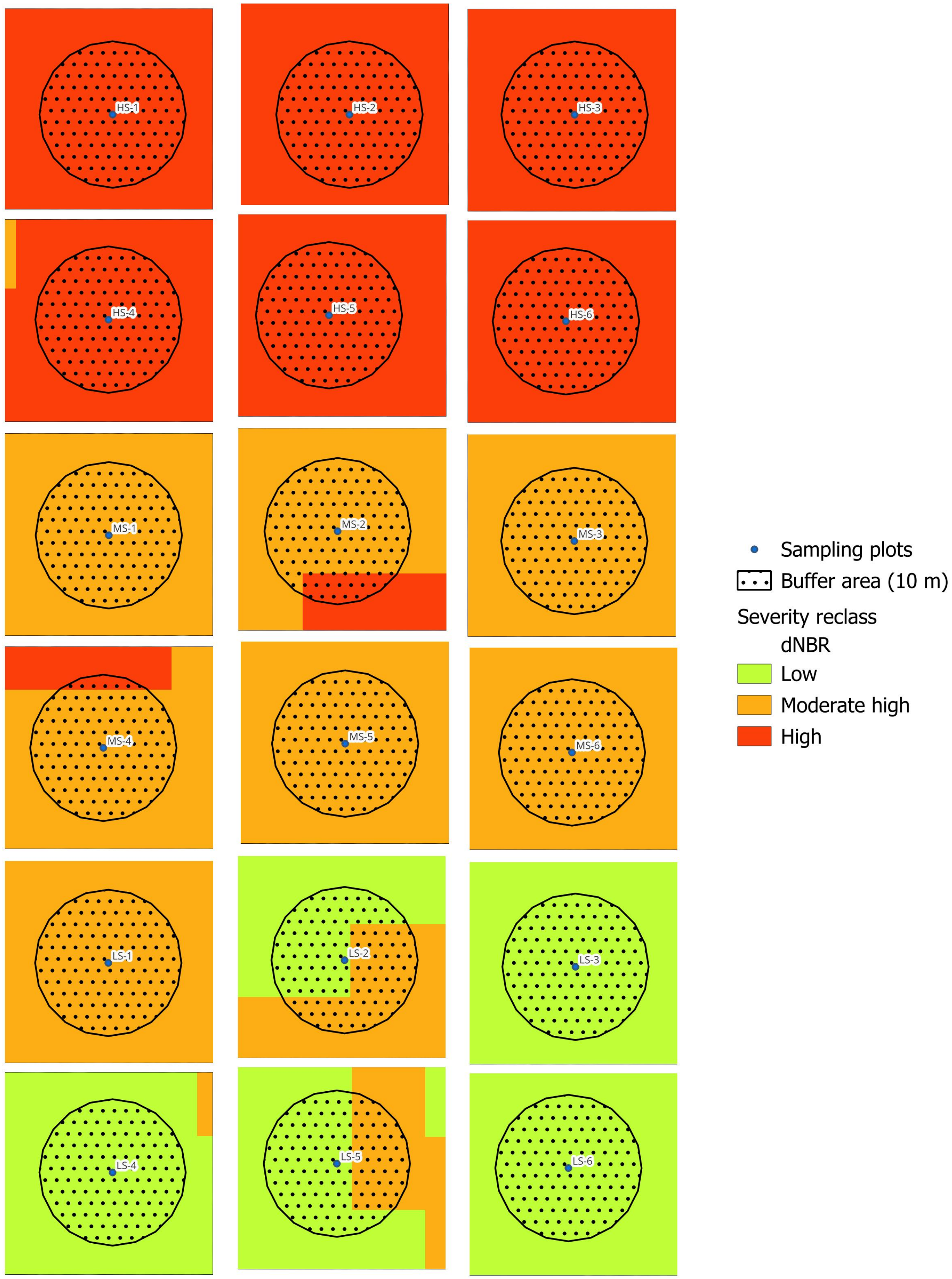

Figure S6. Sampling sites on index dNBR index reclassifies fire severity based on our threshold

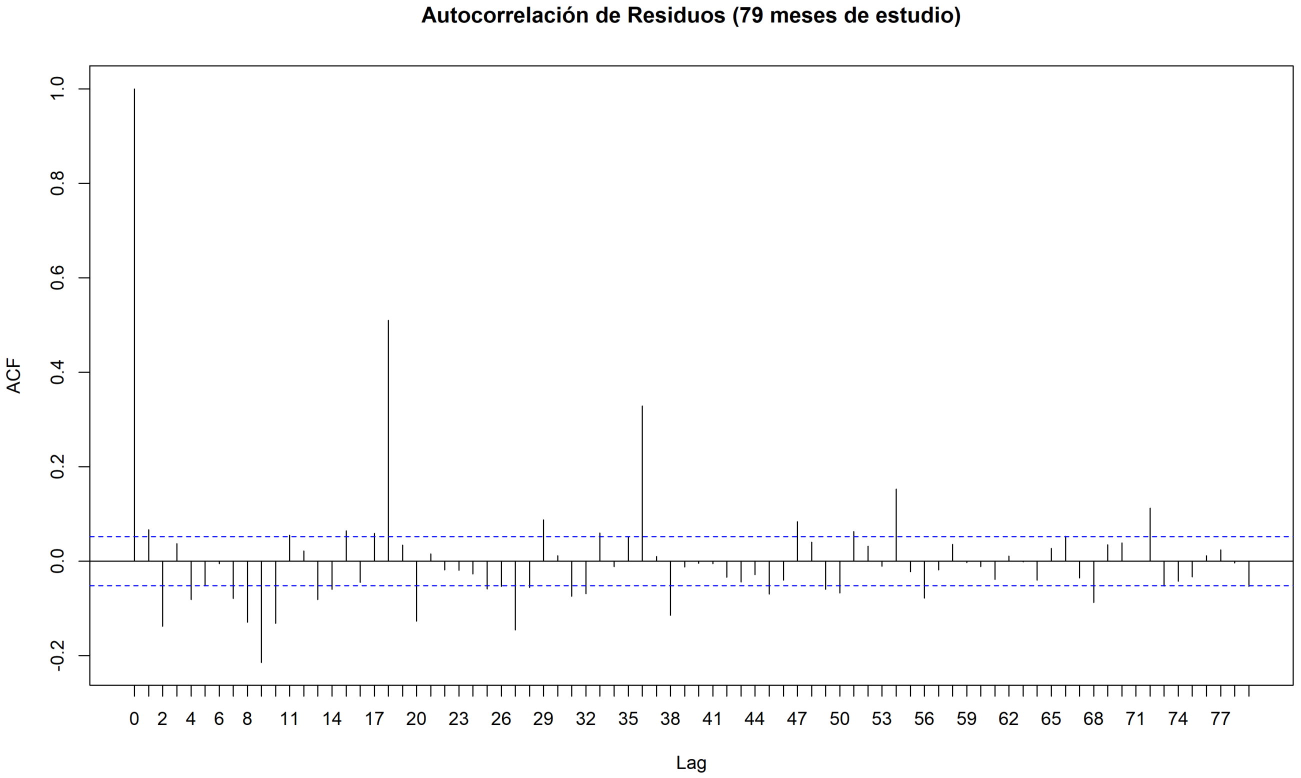

Figure S7. ACF NDVI lineal mixed model Residuals
